# Phase II study of maintenance trifluridine/tipiracil (TAS-102) plus bevacizumab after induction chemotherapy in metastatic colorectal cancer

**DOI:** 10.1093/oncolo/oyag067

**Published:** 2026-02-28

**Authors:** Katsuya Ota, Mamoru Uemura, Tsukasa Tanida, Ken Nakata, Toshihiro Kudo, Yoshinori Kagawa, Naotsugu Haraguchi, Soichiro Minami, Yusuke Matsuura, Hirofumi Ota, Masayuki Hiraki, Masayoshi Yasui, Hidekazu Takahashi, Kazuya Iwamoto, Naohiro Nishida, Taishi Hata, Yujiro Nishizawa, Mutsumi Fukunaga, Takamichi Komori, Yusuke Takahashi, Shinji Tokuyama, Hiroshi Tamagawa, Atsushi Naito, Terukazu Yoshihara, Rei Suzuki, Satoshi Sugimoto, Masakazu Miyake, Nobuo Takiguchi, Mitsuyoshi Tei, Koki Tamai, Akio Fukada, Takayuki Ogino, Norikatsu Miyoshi, Taroh Satoh, Hirofumi Yamamoto, Kohei Murata, Yuichiro Doki, Hidetoshi Eguchi

**Affiliations:** Department of Gastroenterological Surgery, Kindai University Nara Hospital, Ikoma City, Nara, 630-0293, Japan; Department of Gastroenterological Surgery, Graduate School of medicine, The University of Osaka, Suita City, Osaka, 565-0871, Japan; Department of Gastroenterological Surgery, Higashiosaka City Medical Center, Higashiosaka City, Osaka, 578-0947, Japan; Department of Gastroenterological Surgery, Higashiosaka City Medical Center, Higashiosaka City, Osaka, 578-0947, Japan; Department of Medical Oncology, Osaka International Cancer Institute, Osaka City, Osaka, 541-8567, Japan; Department of Gastroenterological Surgery, Osaka International Cancer Institute, Osaka City, Osaka, 541-8567, Japan; Department of Gastroenterological Surgery, Kindai University Nara Hospital, Ikoma City, Nara, 630-0293, Japan; Department of Gastroenterological Surgery, Kindai University Nara Hospital, Ikoma City, Nara, 630-0293, Japan; Department of Gastroenterological Surgery, Ikeda City Hospital, Ikeda City, Osaka, 563-8510, Japan; Department of Gastroenterological Surgery, Ikeda City Hospital, Ikeda City, Osaka, 563-8510, Japan; Department of Gastroenterological Surgery, Kansai Rosai Hospital, Amagasaki City, Hyogo, 660-8511, Japan; Department of Gastroenterological Surgery, Kansai Rosai Hospital, Amagasaki City, Hyogo, 660-8511, Japan; Department of Gastroenterological Surgery, Osaka International Medical & Science Center, Osaka Keisatsu Hospital, Osaka City, Osaka, 543-0035, Japan; Department of Gastroenterological Surgery, Osaka International Medical & Science Center, Osaka Keisatsu Hospital, Osaka City, Osaka, 543-0035, Japan; Department of Medical Oncology, Tokai University School of Medicine, Isehara City, Kanagawa, 259-1193, Japan; Department of Gastroenterological Surgery, Osaka General Medical Center, Osaka City, Osaka, 558-8558, Japan; Department of Gastroenterological Surgery, Osaka General Medical Center, Osaka City, Osaka, 558-8558, Japan; Department of Gastroenterological Surgery, Hyogo Prefectural Nishinomiya Hospital, Nishinomiya City, Hyogo, 662-0918, Japan; Department of Gastroenterological Surgery, Hyogo Prefectural Nishinomiya Hospital, Nishinomiya City, Hyogo, 662-0918, Japan; Department of Surgery, NHO Osaka National Hospital, Osaka City, Osaka, 540-0006, Japan; Department of Surgery, NHO Osaka National Hospital, Osaka City, Osaka, 540-0006, Japan; Department of Gastroenterological Surgery, Otemae Hospital, Osaka City, Osaka, 540-0008, Japan; Department of Gastroenterological Surgery, Sakai City Medical Center, Sakai City, Osaka, 593-8304, Japan; Department of Gastroenterological Surgery, Sakai City Medical Center, Sakai City, Osaka, 593-8304, Japan; Department of Gastroenterological Surgery, Saiseikai Senri Hospital, Suita City, Osaka, 565-0862Japan; Department of Gastroenterological Surgery, Saiseikai Senri Hospital, Suita City, Osaka, 565-0862Japan; Department of Gastroenterological Surgery, Rinku General Medical Center, Izumisano City, Osaka, 598-8577, Japan; Department of Gastroenterological Surgery, Rinku General Medical Center, Izumisano City, Osaka, 598-8577, Japan; Department of Surgery, Osaka Rosai Hospital, Sakai City, Osaka, 591-8025, Japan; Department of Surgery, Osaka Rosai Hospital, Sakai City, Osaka, 591-8025, Japan; Department of Gastroenterological Surgery, Graduate School of medicine, The University of Osaka, Suita City, Osaka, 565-0871, Japan; Department of Gastroenterological Surgery, Graduate School of medicine, The University of Osaka, Suita City, Osaka, 565-0871, Japan; Department of Gastroenterological Surgery, Graduate School of medicine, The University of Osaka, Suita City, Osaka, 565-0871, Japan; Department of Gastroenterological Surgery, Graduate School of medicine, The University of Osaka, Suita City, Osaka, 565-0871, Japan; Department of Gastroenterological Surgery, Graduate School of medicine, The University of Osaka, Suita City, Osaka, 565-0871, Japan; Department of Gastroenterological Surgery, Kansai Rosai Hospital, Amagasaki City, Hyogo, 660-8511, Japan; Department of Gastroenterological Surgery, Graduate School of medicine, The University of Osaka, Suita City, Osaka, 565-0871, Japan; Department of Gastroenterological Surgery, Graduate School of medicine, The University of Osaka, Suita City, Osaka, 565-0871, Japan

**Keywords:** maintenance therapy, colorectal cancer, TAS-102, bevacizumab, oxaliplatin

## Abstract

**Background:**

First-line chemotherapy with maintenance therapy is expected to be well-tolerated and improve survival in patients with metastatic colorectal cancer (mCRC). This study evaluated the efficacy and safety of trifluridine/tipiracil (TAS-102) plus bevacizumab (Bev) as maintenance therapy for mCRC, omitting both oxaliplatin and fluoropyrimidines.

**Materials and Methods:**

Patients with untreated mCRC initially received induction chemotherapy with fluoropyrimidine, oxaliplatin plus bevacizumab for 3-4 months. After achieving stable disease or better on imaging, 52 patients transitioned to maintenance therapy with TAS+Bev: TAS-102 (35 mg/m^2^, twice daily on days 1-5 and 8-12) plus Bev (5.0 mg/kg on Days 1 and 15, intravenously). Progression-free survival 1 (PFS1), defined as the time from the start of maintenance therapy to disease progression or death, was evaluated as the primary endpoint.

**Results:**

The median cumulative dose of oxaliplatin during induction was 529 mg/m^2^. Median PFS1 during TAS+Bev maintenance was 8.7 months (95% CI: 5.5-12.3). The response rate and disease control rate during maintenance were 59.6% and 94.2%, respectively. Oxaliplatin reintroduction was feasible in 53.8% (28/52) of patient with a median total first-line chemotherapy duration was 16.2 months (95% CI: 14.5-20.6). Safety outcomes, including dose intensity and adverse events, were acceptable.

**Conclusion:**

This strategy of switching to first-line maintenance therapy with TAS+Bev demonstrated promising efficacy and safety. This strategy effectively avoided cumulative neurotoxicity, preserved quality of life and enabled reintroduction of oxaliplatin, suggesting it may represent a promising alternative strategy for patients ineligible for prolonged oxaliplatin-based treatment. Overall survival analysis is currently ongoing. [UMIN Trial ID: UMIN000031317]

Lessons Learned

- This study highlights the novel strategy involving a complete switch-first-line maintenance therapy with TAS-102 plus bevacizumab, omitting both oxaliplatin and fluoropyrimidines. By avoiding neurotoxicity while maintaining tumor control and quality of life, this regimen challenges the conventional norms for maintenance therapy, potentially serving as a new standard for patients ineligible for prolonged oxaliplatin use.
- The two-step registration ensured patient homogeneity and operational feasibility in a multicenter setting. This structured design may be useful for future investigator-initiated trials.

## Trial information

- UMIN trial ID: UMIN000031317
- Principal Investigators: Katsuya Ota https://orcid.org/0000-0003-4897-7032
- Sponsor: None
- IRB Approved: Yes

**Table:** 

**Trial information**	
**Disease**	Colorectal cancer
**Stage of disease/treatment**	Metastatic/advanced
**Prior therapy**	No prior systemic therapy before induction chemotherapy
**Type of study**	Phase II, single arm
**Primary endpoints**	Progression-free survival 1 (PFS1)
**Secondary endpoints**	Overall survival, response rate, disease control rate, oxaliplatin reintroduction rate and safety
**Additional details of endpoints or study design** This single-arm, multicenter phase II trial was conducted by Multicenter Clinical Study Group of The University of Osaka, Colorectal Cancer Treatment Group. This trial was registered at the University Hospital Medical Information Network (UMIN) Clinical Trials Registry as UMIN000031317 on February 16, 2018. This study investigated the efficacy and safety of maintenance therapy with trifluridine/tipiracil (TAS-102) plus bevacizumab (TAS+Bev) as the first-line treatment for patients with unresectable CRC after initial induction chemotherapy (Figure 1, CONSORT diagram). The primary endpoint was progression-free survival 1 (PFS1), defined as the time from the start of maintenance therapy to disease progression or death from any cause (Figure 2). The secondary endpoints were overall survival (OS), response rate (RR), disease control rate (DCR), oxaliplatin reintroduction rate, and treatment-related adverse events (graded according to CTCAE v5.0). The patient cohort included adults with histologically confirmed metastatic colorectal cancer (mCRC) who were enrolled prior to induction chemotherapy with fluoropyrimidine, oxaliplatin, and bevacizumab to ensure quality control. The patients must have achieved disease control (Complete response; CR, Partial response; PR, or Stable disease; SD based on Response Evaluation Criteria in Solid Tumors: RECIST version 1.1) after 4-6 cycles of capecitabine, oxaliplatin, and bevacizumab (CapeOX+Bev) or 6-8 cycles of oxaliplatin, fluoropyrimidine, and bevacizumab (FOLFOX+Bev) without exceeding a cumulative oxaliplatin dose of 680 mg/m² (or 1000 mg/patient). After induction, patients were re-evaluated for eligibility in a second registration step before starting maintenance therapy. At this transition, the same eligibility (11 items) and exclusion (19 items) criteria as those used at initial registration were re-confirmed (UMIN000031317), and no additional criteria were introduced. Only patients who achieved CR, PR, or SD based on RECIST version 1.1 and continued to meet all eligibility requirements proceeded to the maintenance phase. The maintenance therapy consisted of oral TAS-102 and intravenous bevacizumab, administered until radiologic/clinical progression or intolerable toxicity. Tumor assessments were conducted every 8 weeks per RECIST v1.1 criteria. The trial is powered of 80% for detecting a pre-specified improvement in PFS, with a two-sided alpha of 0.05. The estimated sample size was 52 patients. To account for potential dropouts or ineligibility, the target enrollment was set at 55 patients. Ultimately, 52 patients were included in the final analysis. The Data and Safety Monitoring Committee of The University of Osaka independently reviewed the efficacy and safety data obtained from the present study, as well as monitored protocol compliance, and safety progress. The Protocol Review Committee approved this study protocol on November 7, 2017. Approval was obtained from the Institutional Review Board before starting patient enrollment at each institution. The study was conducted in accordance with the guidelines of the Declaration of Helsinki and the International Conference on Harmonization E6 Good Clinical Practice. The present study has no support or funding. The ethical committee or institutional review committee at each site approved the protocol before the initiation of the study. All patients were required to sign a written informed consent form.	

**Figure 1.**
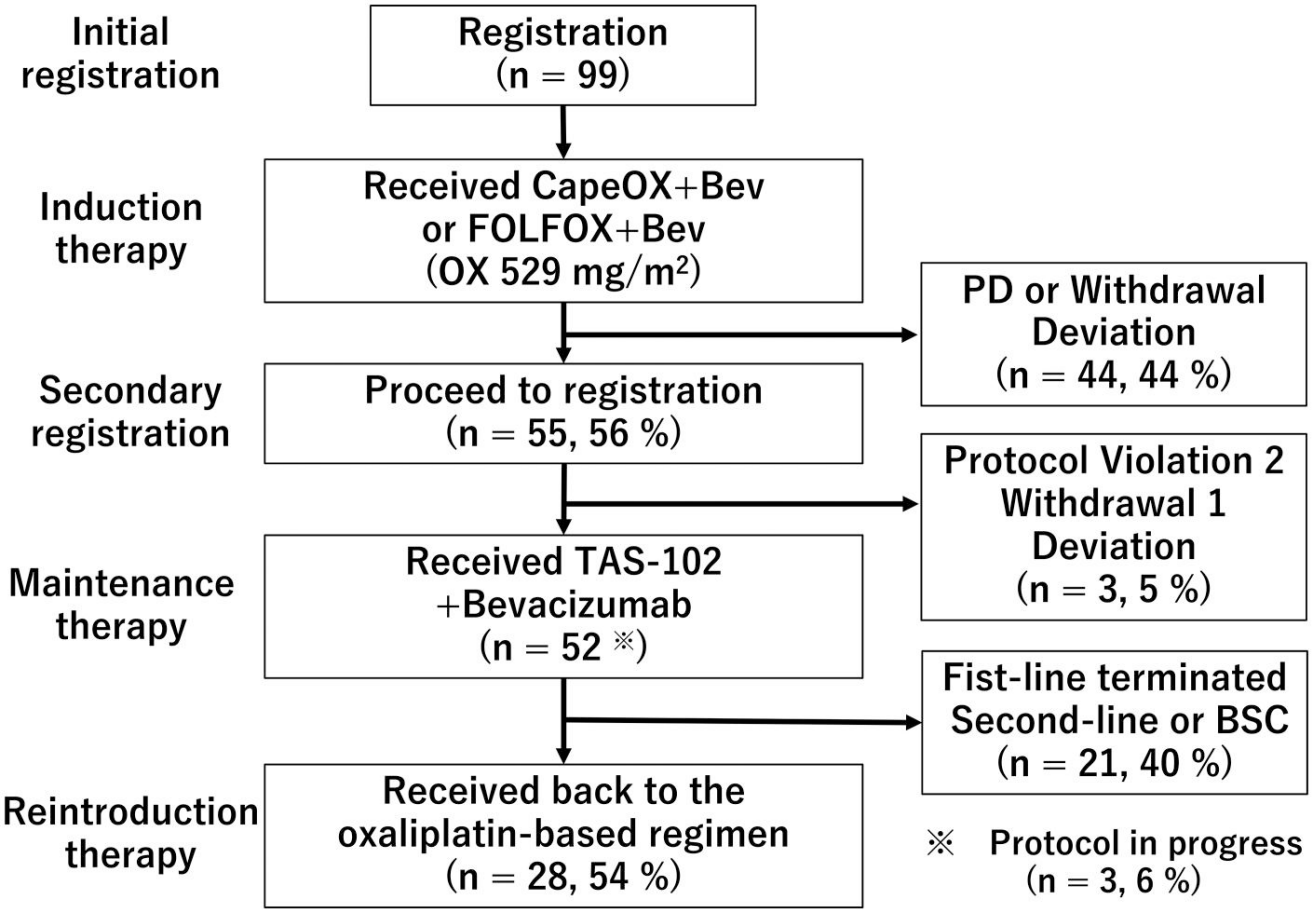
CONSORT diagram of the Switch Maintenance Study. CapeOX, capecitabine and oxaliplatin; Bev, bevacizumab; FOLFOX, folinic acid, fluorouracil and oxaliplatin; PD, progression disease; TAS+Bev, TAS-102 and bevacizumab; BSC, best supportive care.

**Figure 2.**
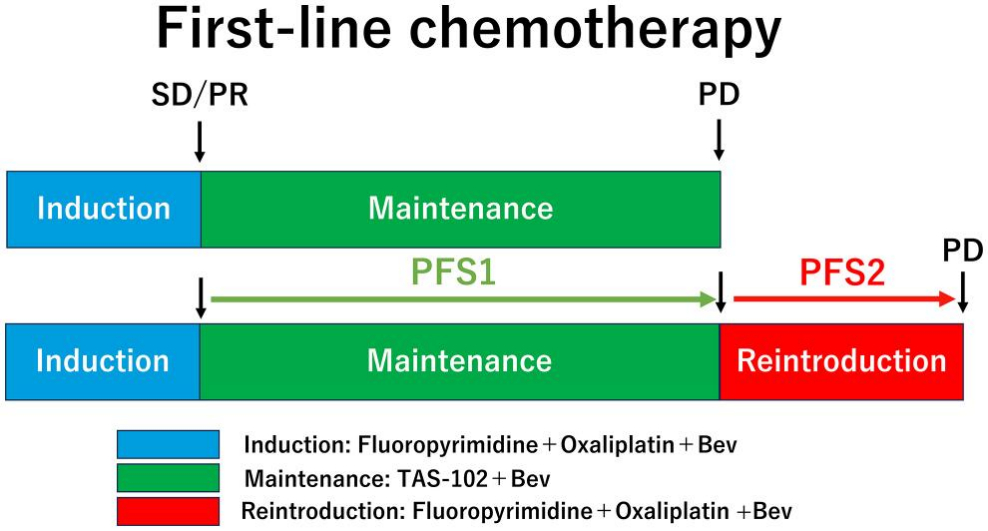
Definition of induction, maintenance therapy and reintroduction during first-line chemotherapy. PFS1 was defined as the time from the start of maintenance therapy to disease progression or death, and PFS2 as the time from the start of reintroduction therapy to subsequent progression or death. SD, stable disease; PR, partial response; PD, progression disease; PFS, progression-free survival.

**Table:** 

**Drug information**	
**Generic/working name**	TAS-102 (Lonsurf/New drug), Bevacizumab (Avastin or Generic/Bevacizumab)
**Company name**	Taiho Pharmaceutical, Chugai Pharmaceutical or Generic
**Drug type**	Chemotherapy
**Drug class**	TAS-102 (Trifluridine-Tipiracil Hydrochloride Mixture) is a combination of a thymidine-based nucleic acid analogue, trifluridine, and a thymidine phosphorylase inhibitor, tipiracil hydrochloride. Bevacizumab (anti-VEGF) is a humanized anti-VEGF monoclonal antibody which binds to and neutralizes all human VEGF-A isoforms and bioactive proteolytic fragments.
**Dose**	TAS-102 35 mg/m^2^/time, twice a day Bevacizumab venous injection: 5.0 mg/kg
**Route**	TAS-102: oral Bevacizumab: intravenous
**Schedule of administration treatment plan for maintenance therapy**: Patients fulfilling the eligibility criteria during the second registration (main enrollment) will receive maintenance therapy with TAS-102 plus bevacizumab (TAS+Bev) until disease progression (PD). 1. TAS-102 (Trifluridine/Tipiracil Hydrochloride) TAS-102 (Lonsurf) is administered orally at 35 mg/m² per dose, twice daily (morning and evening, after meals). Within a 28-day cycle, the drug is given on days 1-5 and 8-12 followed by a 14-days rest period (days 15-28). The drug is formulated into 15-mg and 20-mg tablets. The total daily dose is determined by body surface area. 2. Bevacizumab Bevacizumab (Avastin or Generic) is administered at a dose of 5.0 mg/kg via intravenous infusion on days 1 and 15 of a 28-day cycle. The first infusion is administered over 90 minutes and the second is given over 60 minutes. If no infusion-related adverse events are observed during the initial administration, the subsequent infusions may be shortened to 30 minutes. A saline flush should be administered after infusion to ensure complete drug delivery.	

**Table:** 

**Patient characteristics**	
**Number of patients, male**	30
**Number of patients, female**	22
**Stage**	Stage IV, *n* = 40 (78.0%); recurrent disease, *n* = 12 (22.0%)
**Number of prior systemic therapies: median (range)**	Induction chemotherapy; CapeOX+Bev, *n* = 49 (94.2%); FOLFOX+Bev: 3 (5.8%); median number of 5 courses (3-8); median oxaliplatin infusion, 529 mg/m^2^ (range: 347-781 mg/m^2^) Clinical response after induction chemotherapy (CR/PR/SD): CR, *n* = 0; PR, *n* = 30 (57.7%); SD *n* = 22 (42.3%)
**Performance status: ECOG 0 or 1**	ECOG 0: *n* = 45 (90.4%), ECOG 1: *n* = 7 (13.5%)
**Performance status: ECOG 2 or above**	0
**Tumor Location:**	Right-sided (cecum, ascending colon, or transverse colon): *n* = 14 (26.9%); left-sided (descending colon, sigmoid colon, or rectum): *n* = 38 (73.1%)
**RAS-wild-type/RAS-mutant-type**	Wild-type, *n* = 18 (34.6%)/mutant-type: *n* = 34 (65.4%)
**Cancer types or histologic subtypes**	Tubular adenocarcinoma: *n* = 48 (92.3%); poorly differentiated adenocarcinoma: *n* = 2 (3.8%); mucinous adenocarcinoma: *n* = 2 (3.8%)

**Table:** 

**Primary assessment method**	
**Title**	Progression-free survival 1 (PFS1)
**Number of patients screened**	55
**Number of patients enrolled**	52
**(Median) Duration assessments PFS1**	8.7 months (95% CI: 5.5-12.3)
**Outcome notes** In the survival analysis, 3 patients were excluded due to lack of baseline imaging, violation of induction chemotherapy protocols, and withdrawal of participation (*n* = 1 each), resulting in 52 patients in the final analysis. The Kaplan–Meier curve for PFS1 during the TAS+Bev period in these 52 patients is shown in Figure 3A. The data cutoff was set at December 31, 2024, which included 3 patients who were still receiving treatment. Among the 49 patients, 40 experienced disease progression and 9 patients had not progressed at the data cutoff. Of these 9 patients, 6 discontinued due to toxicity, 2 died without progression, and 1 refused further treatment. The median PFS1 of the 52 patients was 8.7 months (95% CI: 5.5-12.3). Patients received a median of 7 TAS+Bev cycles (range, 1-20).	

**Table:** 

**Secondary assessment method**	
**Title**	Overall survival, response rate, disease control rate, oxaliplatin reintroduction rate, and treatment-related adverse events during TAS+Bev administration.
**Number of patients screened**	52
**Number of patients enrolled**	52
**(Median) Duration assessments OS**	37.1 months (95% CI: 28.5-55.2)
**Number of patients evaluable for efficacy (as per RECIST 1.1)** **Outcome notes**	Overall response summary: *n* = 52; CR, *n* = 4 (7.7%); PR, *n* = 27 (51.9%); SD, *n* = 18 (34.6%); NE, *n* = 2 (3.8%); PD, *n* = 1 (1.9%) The best response during the TAS+Bev period, demonstrated best percentage changes in tumor diameter. The response rate (RR) was 59.6%, disease control rate (DCR) was 94.2%. There was no significant difference in the best response between patients with right-sided tumors (cecum, ascending colon, or transverse colon) and left-sided tumors (descending colon, sigmoid colon, or rectum), nor between those with RAS-wild and RAS-mutant tumors.
**Number of patients evaluated for toxicity (as per CTCAE v5.0)**	52 (see adverse events)
**Outcome notes** The Kaplan–Meier curves for OS in the 52 patients from the start of TAS+Bev therapy to death are shown in Figure 3B. As 34 patients (65.4%) were still alive at the time of analysis, the data cutoff was set on December 31, 2024. The median OS overall was 37.1 months (95% CI: 28.5-55.2). Oxaliplatin was reintroduced in 53.8% (28 patients), with a median cumulative reintroduction dose of 396 mg/m^2^ (range: 83-1899 mg/m^2^). Progression-free survival 2 (PFS2), defined as the time from the start of reintroduction therapy to disease progression or death from any cause, was 5.6 months (95% CI: 4.3-7.0, Figure 2). At the data cutoff, three patients were still receiving treatment and were censored, not excluded, from the analysis. Among the 21 patients (40.2%) who did not undergo oxaliplatin reintroduction, the reasons were as follows: TAS-102-related toxicity (*n* = 7), oxaliplatin-related toxicity (*n* = 4), investigator’s decision (*n* = 1), transition to best supportive care (BSC, *n* = 5), death due to other causes (*n* = 2), surgery prioritized (*n* = 1), and refusal of further treatment (*n* = 1). Of these, 12 patients, those with TAS-102-related toxicity, oxaliplatin-related toxicity and investigator’s decision, subsequently received second-line chemotherapy (capecitabine plus Bev [Cape+Bev], *n* = 3; re-challenged TAS+Bev, *n* = 3; TAS-102 monotherapy, *n* = 1; capecitabine plus irinotecan+Bev [CapeIRI+Bev], *n* = 2; S-1 plus irinotecan +Bev [IRIS+Bev], *n* = 1; FOLFIRI plus ramucirumab [FOLFIRI+RAM], *n* = 1; S-1 plus paclitaxel [S-1+PTX], *n* = 1). The median total first-line chemotherapy duration (ie, from the start of induction therapy until disease progression after reintroduction or the end of protocol therapy) was 16.2 months (95% CI: 14.5-20.6, Figure 4).	

**Figure 3.**
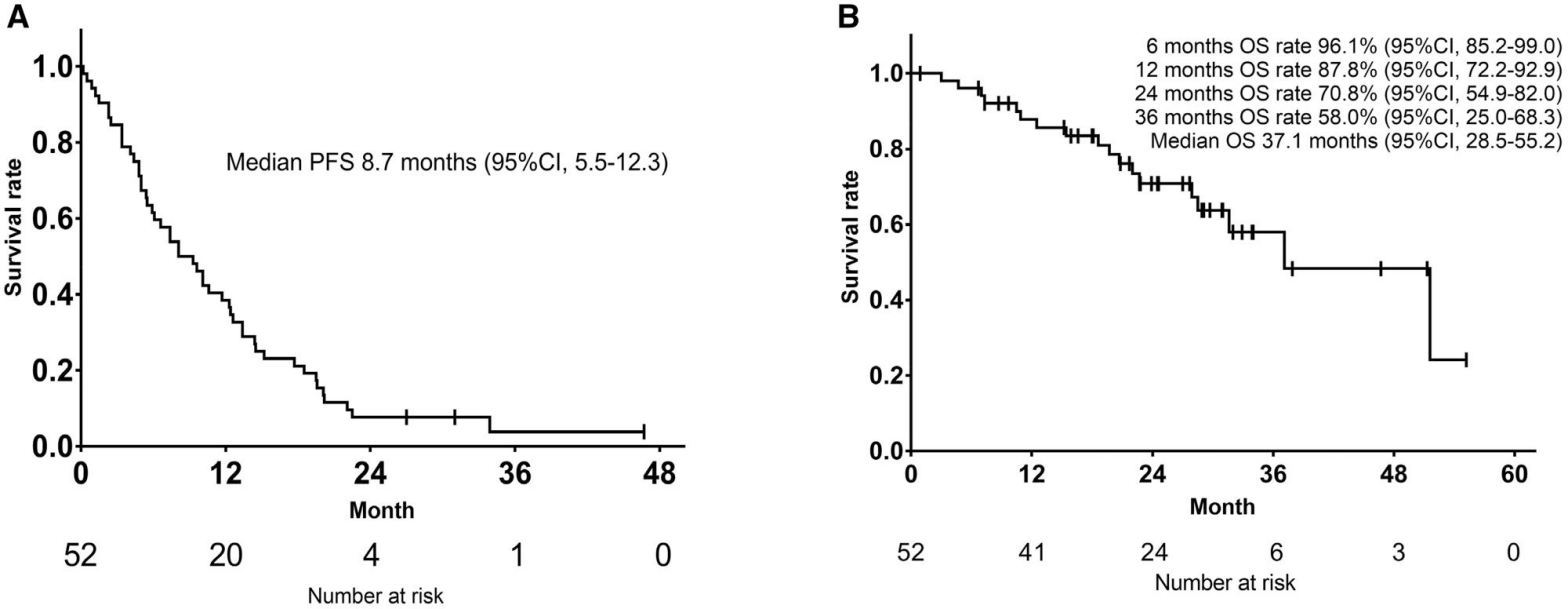
A. Kaplan–Meier curve of progression free survival as primary endpoint demonstrating disease control durability with TAS-102 plus bevacizumab (*n* = 52). B. Kaplan–Meier curve of overall survival (*n* = 52). At the data cutoff (December 31, 2024), 3 patients were still receiving treatment and were censored, not excluded, from the analysis. Number at risk are shown below each curve. CI, confidence interval; PFS, progression free survival; OS, overall survival.

**Figure 4.**
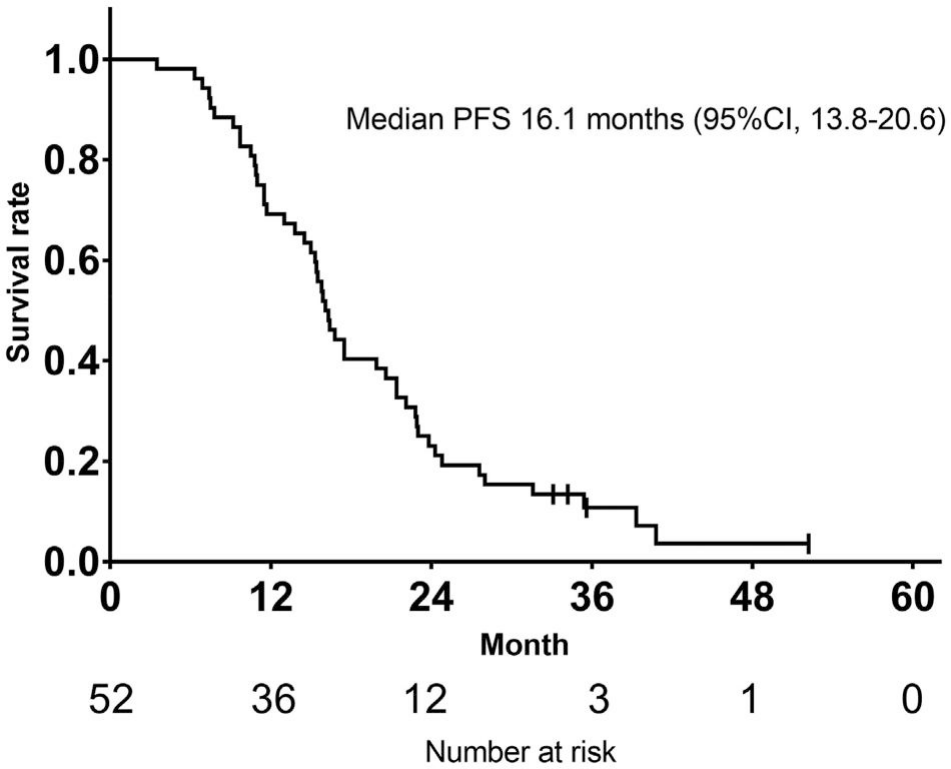
Kaplan–Meier curve of progression free survival at duration of total first-line treatment, including induction, TAS+Bev maintenance, and oxaliplatin reintroduction (*n* = 52). Number at risk are shown below each curve. CI, confidence interval. PFS, progression free survival.

## General toxicity profile

All patients were evaluable for safety using CTCAE v5.0 (Table 1). The observed major grade 3 or 4 hematologic toxicities were neutropenia (*n* = 38, 73%), white blood cell decreased (*n* = 18, 35%), platelet count decreased (*n* = 5, 10%) and anemia (*n* = 4, 8%). The grade 3 non-hematologic toxicities included hypertension (*n* = 4, 8%), proteinuria (*n* = 4, 8%), fatigue (*n* = 3, 6%) and fever (*n* = 2, 4%). There was no grade 3 or higher peripheral sensory neuropathy (any-grade incidence, *n* = 16, 31%) or thromboembolic event (any-grade incidence, *n* = 1, 2%). There was no treatment-related death.

**Table 1.** Adverse events (AEs).

Adverse events All cycles							
Name	NC/NA	1	2	3	4	5	All grades
**White blood cell decreased**	33%	4%	29%	31%	4%	0	67%
**Neutrophil count decreased**	15%	0	12%	48%	25%	0	85%
**Anemia**	60%	21%	12%	8%	0	0	40%
**Platelet count decreased**	65%	17%	8%	8%	2%	0	35%
**Peripheral sensory neuropathy**	69%	15%	15%	0	0	0	31%
**Fever**	83%	10%	4%	4%	0	0	27%
**Hypertension**	73%	6%	13%	8%	0	0	27%
**Proteinuria**	73%	10%	10%	8%	0	0	27%
**Fatigue**	50%	25%	19%	6%	0	0	50%
**Diarrhea**	65%	29%	4%	2%	0	0	35%
**Nausea**	87%	12%	0	2%	0	0	13%
**Mucositis oral**	90%	6%	0	4%	0	0	10%
**Lung infection**	92%	0	4%	4%	0	0	8%
**Urinary tract obstruction**	98%	0	0	2%	0	0	2%
**Bronchopulmonary hemorrhage**	98%	0	0	2%	0	0	2%
**Thromboembolic event**	98%	0	2%	0	0	0	2%

NC/NA, no change from baseline/no adverse event.

**Table:** 

Assessment, analysis, and discussion	
Completion	Study completed
Investigator’s assessment	Active and should be pursued further

## Discussion

This prospective, multicenter study evaluated the efficacy and safety of a complete switch to first-line maintenance therapy (TAS+Bev) after induction chemotherapy (fluoropyrimidine plus oxaliplatin) in previously untreated patients with mCRC. The study was completed as planned. The strategy specifically omits both oxaliplatin and fluoropyrimidines during the maintenance phase, aiming to reduce cumulative toxicity while maintaining disease control. Maintenance therapy with TAS+Bev achieved a median PFS1 of 8.7 months, with a high DCR of 94.2% and RR of 59.6%. Oxaliplatin reintroduced in 53.8% of patients after maintenance therapy. Total first-line treatment duration was 16.2 months. The median OS among 52 patients was 37.1 months.

These findings suggest that the ability to sustain long-term maintenance treatment may not be easily predicted by conventional baseline factors, thereby needing further investigation into novel clinical or molecular predictors. Nevertheless, first-line maintenance therapy with TAS+Bev can effectively bridge induction and reintroduction phases while preserving therapeutic efficacy. This approach circumvents cumulative peripheral neuropathy, a common and often dose-limiting toxicity in oxaliplatin-containing chemotherapy that negatively affects quality of life and frequently necessitates early treatment discontinuation.^1^ By transitioning to TAS+Bev, patients were able to avoid neurotoxicity while remaining on active therapy.

Previous studies have explored similar de-escalation strategies. The GERCOR OPTIMOX1 trial introduced a “Stop-and-Go” approach with intermittent oxaliplatin administration, which prolonged the treatment duration without compromising efficacy.^2^ The OPTIMOX2 trial later emphasized the importance of maintenance therapy by demonstrating that complete treatment cessation had inferior outcomes.^3^ Likewise, the CAIRO3^4^ and CCOG0902^5^ studies supported maintenance strategies with capecitabine plus bevacizumab after induction therapy. Collectively, these findings highlight the clinical value of structured maintenance approaches. In refractory mCRC, the efficacy of TAS-102, a combination of trifluridine and tipiracil hydrochloride, has been established^9^. Moreover, its combination with bevacizumab has demonstrated synergistic efficacy in the C-TASK FORCE^6^ and SUNLIGHT^7^ trials, leading to improved PFS and OS compared to TAS-102 alone. In the TASCO1 study, this regimen was tolerable and effective even among patients unfit for intensive therapy, suggesting its utility beyond late-line settings. Remarkably, the present study revealed several patients are now continuing maintenance therapy beyond 2 years without disease progression. However, due to protocol-defined constraints, observation beyond this period exceeded the data cut-off.

From a biological standpoint, this strategy may also leverage mechanisms of drug sensitivity restoration. Sharma et al. demonstrated that chromatin remodeling plays a role in transient drug tolerance, suggesting that temporal withdrawal of certain agents might resensitize tumor cells to previously used therapies.^8^ Furthermore, drug rechallenges and treatment beyond progression have also been explored as valid therapeutic strategy in drug-resistant cancers.^10^ Our study builds on this foundation by introducing TAS+Bev earlier in the treatment course as a proactive maintenance strategy rather than a salvage option.

The strengths of this study include its prospective, multicenter design and the two-step registration protocol, which ensured that only patients with stable disease or better on imaging after induction were enrolled into the maintenance phase. This rigorous patient selection likely contributed to the robustness of the results. Conversely, in CAIRO3 study, reintroduction of CapeOX+Bev was permitted in both arms. In that study, 61% of patients in the observation group (ie, did not undergo chemotherapy) underwent reintroduction therapy, compared to only 47% in the Capecitabin plus bevacizumab maintenance group.^4^ This difference suggests that maintenance treatment can delay disease progression, consequently reducing the need for the early reintroduction of combination chemotherapy. In contrast, patients in the observation group, who discontinued therapy tended to progress earlier, thereby requiring reintroduction more frequently. These findings support the clinical utility of maintenance therapy in prolonging PFS and minimizing the need for more intensive treatment over time. Using this regimen allowed for the reintroduction of oxaliplatin in 53.8% of patients in this study, which is a higher rate than the Cap+Bev maintenance group.

The limitations of this study must be acknowledged. Despite being a prospective study, selection bias may still be present because patients transitioning to maintenance may have had more favorable prognostic features. The study also did not include a comparator arm, such as capecitabine plus bevacizumab or observation, limiting our ability to assess the relative efficacy of TAS+Bev. Furthermore, since the OS analysis is ongoing, no definitive conclusions regarding survival benefit can be drawn yet. As with other maintenance therapy trials, OS can be influenced by multiple confounding factors, including post-progression treatments and crossover.^4^

In previous clinical trials, approximately 68% to 79% of patients with mCRC transition from second-line to third-line,^11–13^ but when accounting for patients who deviate from first-line treatment protocols or discontinue early, around 40% to 60% of patients treated with multidrug regimens may not reach third- or fourth-line therapy. Thus, agents that are typically reserved for later-line therapy, such as TAS-102, are rarely ever utilized in such populations. Since this strategy switches away from fluoropyrimidine and oxaliplatin during the maintenance phase, key cytotoxic agents can be preserved for future use. Our OS analysis indicated that maintenance therapy with TAS+Bev was not detrimental, and when oxaliplatin was reintroduced, patients experienced notably improved outcomes. This highlights the clinical relevance of a structured treatment strategy that includes planned reintroduction after maintenance therapy. The favorable prognosis of this subgroup suggests that preserved sensitivity to previously administered agents contributes to the success of maintenance therapy. Nevertheless, the efficacy of this “induction–maintenance–reintroduction” strategy needs to be validated in a phase III randomized controlled trial. If proven non-inferior to conventional maintenance therapies and associated with an OS benefit, this strategy could offer a patient-centered, tolerable, and effective alternative to conventional maintenance strategies, especially for those unfit for long-term oxaliplatin-based therapy. In future seeking to replicate these results, a randomized design including active comparators could enhance the robustness of the findings, particularly in terms of OS and patient-reported outcomes. Additionally, future large-scale prospective trials are needed to confirm and establish TAS+Bev as a standard maintenance option, as well as to better define the optimal patient population using molecular or clinical biomarkers.

## Data Availability

The data underlying this article will be shared on reasonable request to the corresponding author.
